# Supplementary figures and images for: Transcriptional downregulation of rhodopsin is associated with desensitization of rods to light-induced damage in a murine model of retinitis pigmentosa
Source: Hum Mol Genet. 2025 Oct 7;34(22):1884–901. doi: 10.1093/hmg/ddaf146 (PMC12581828; doi:10.1093/hmg/ddaf146)

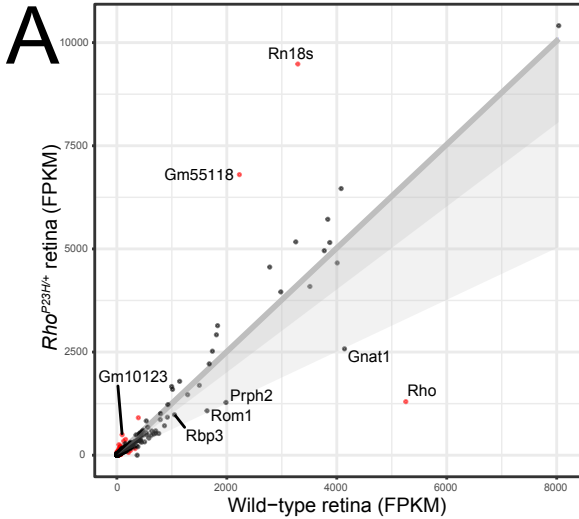

Supplemental Figure S1

Supplement: Supplemental_Figure_S1_RNA-seq_P23H_scatter_plot_ddaf146 [file supplemental_figure_s1_rna-seq_p23h_scatter_plot_ddaf146.pdf]

A

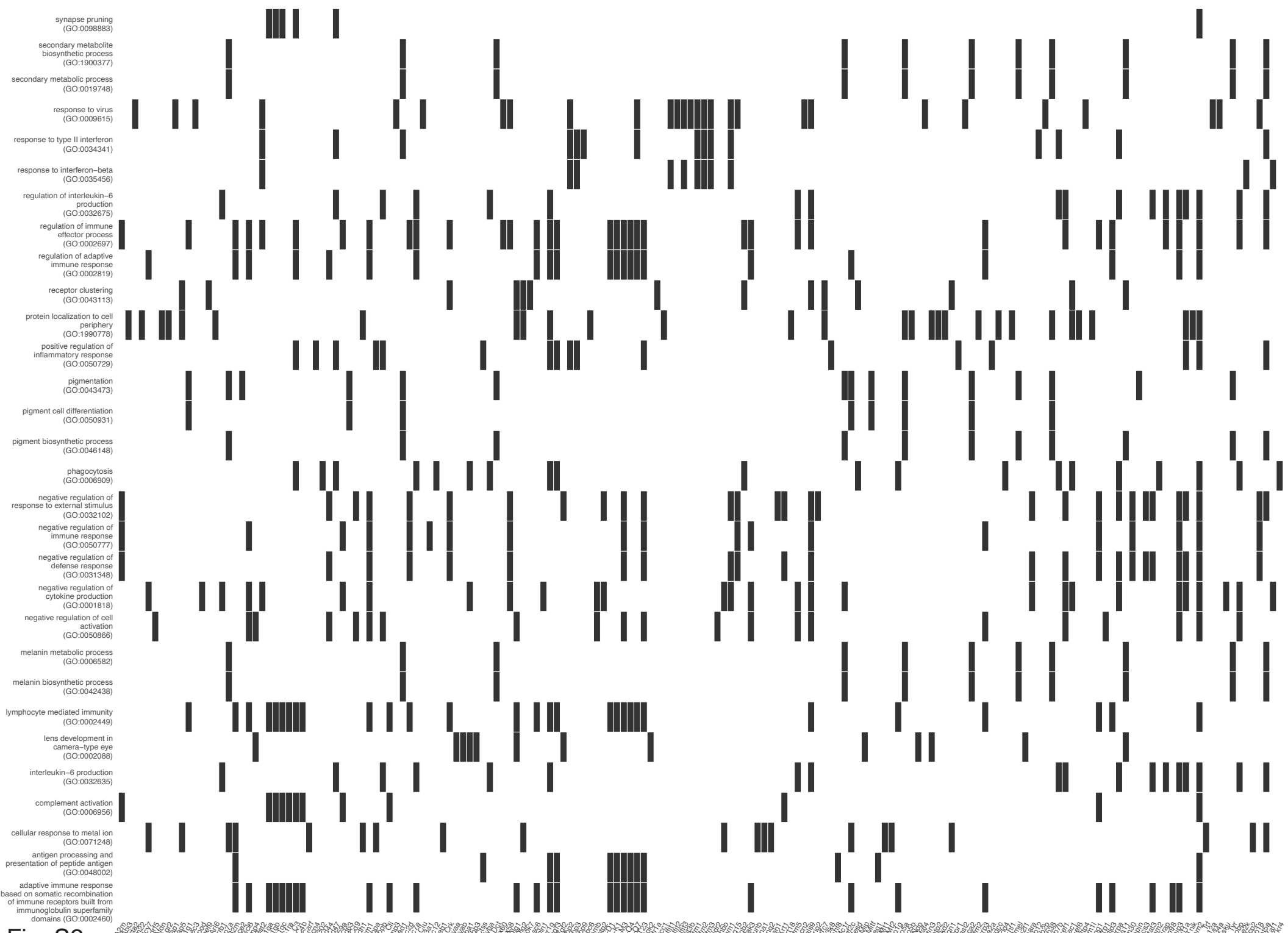

Fig. S2

Supplement: Supplemental_Figure_S2_RNA-seq_Heatmap_for_Q344ter_basic_GO_terms_and_genes_ddaf146 [file supplemental_figure_s2_rna-seq_heatmap_for_q344ter_basic_go_terms_and_genes_ddaf146.pdf]

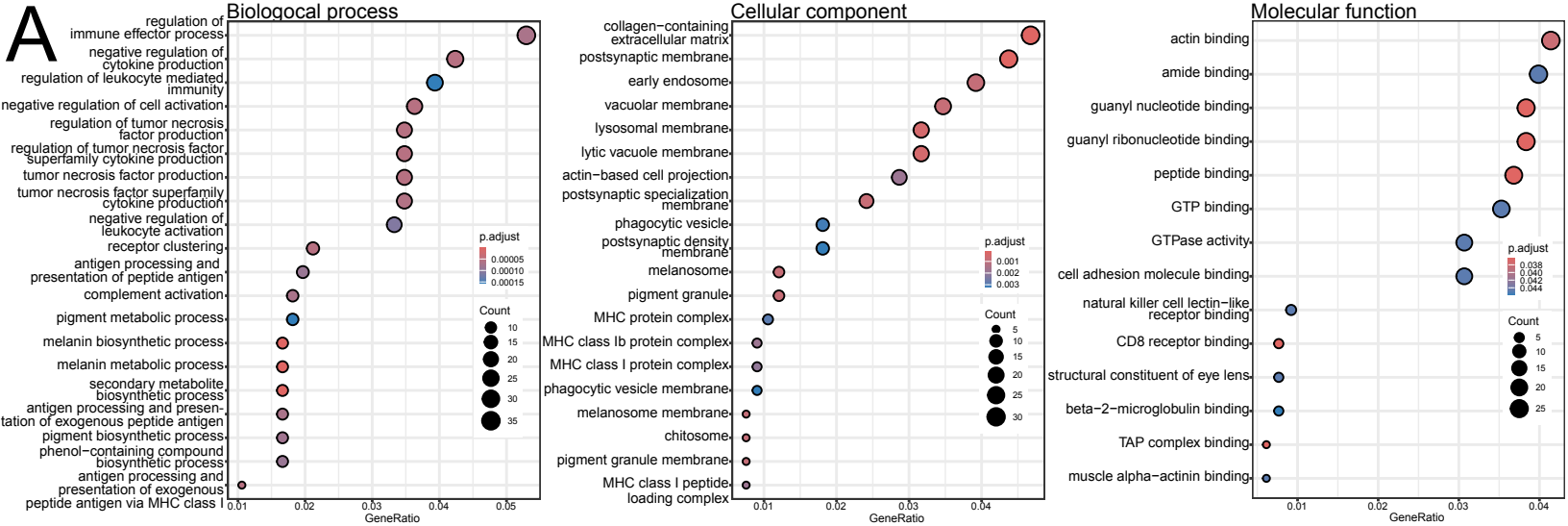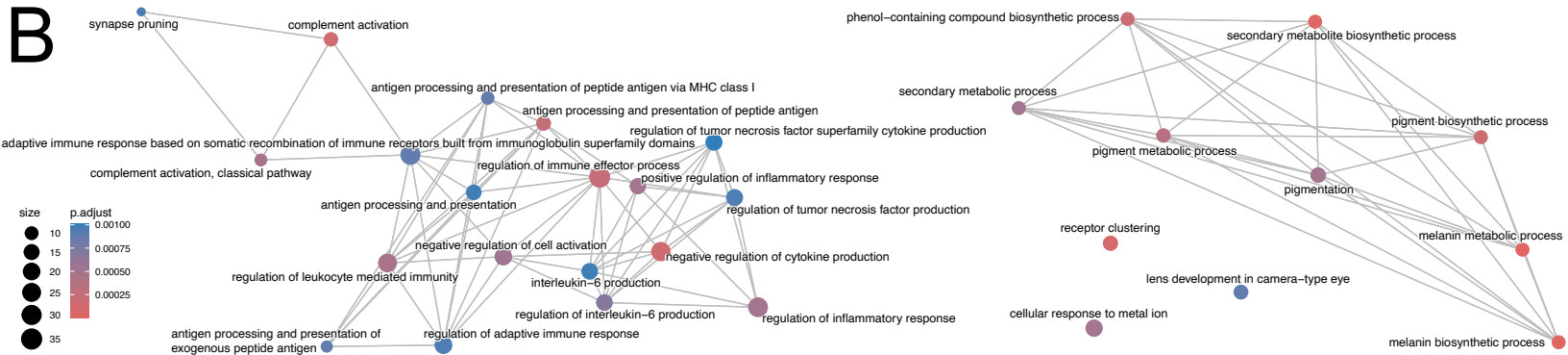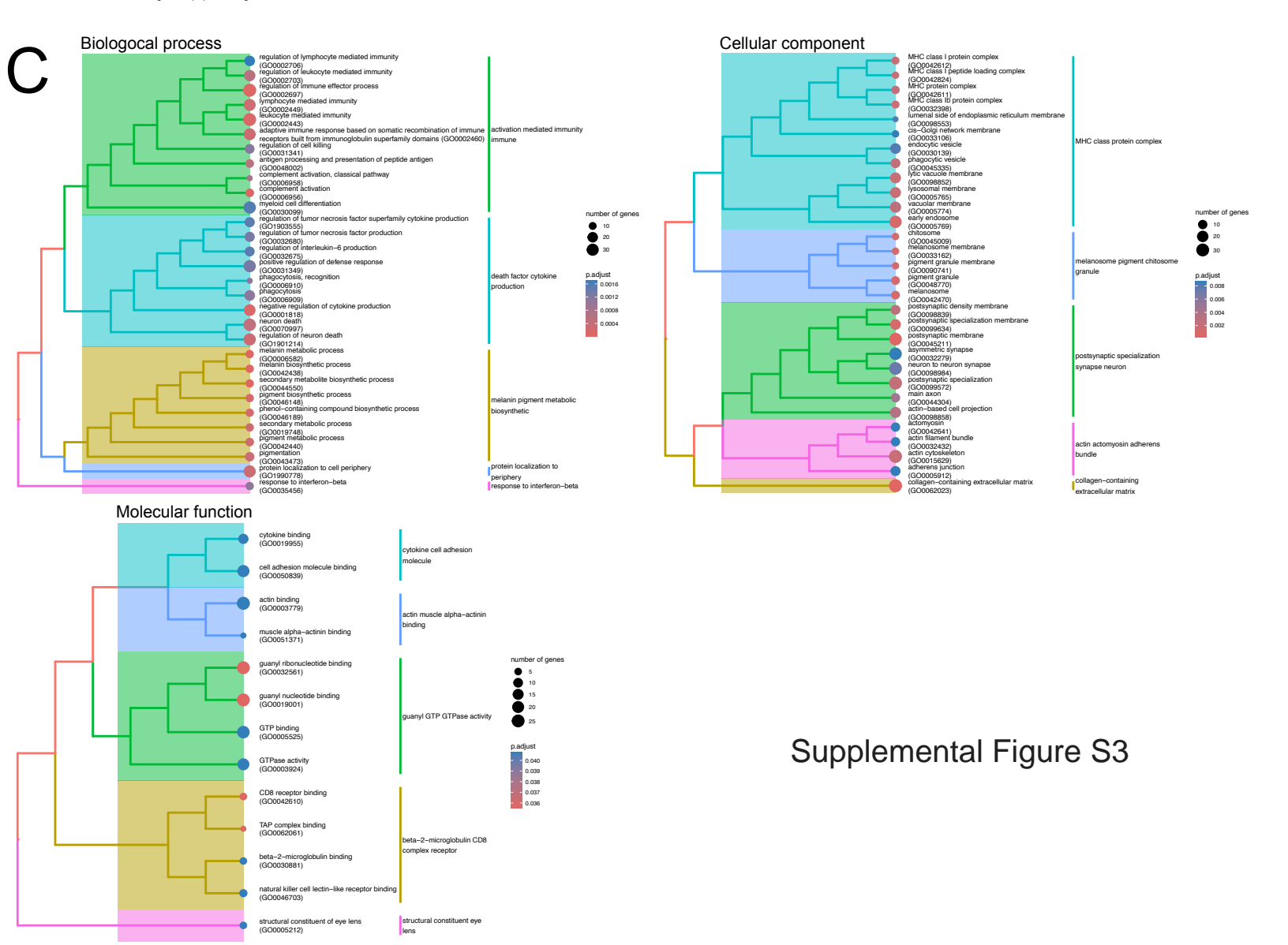

Supplemental Figure S3

Supplement: Supplemental_Figure_S3_Q344X_RNA-seq_basic_071525_ddaf146 [file supplemental_figure_s3_q344x_rna-seq_basic_071525_ddaf146.pdf]

A

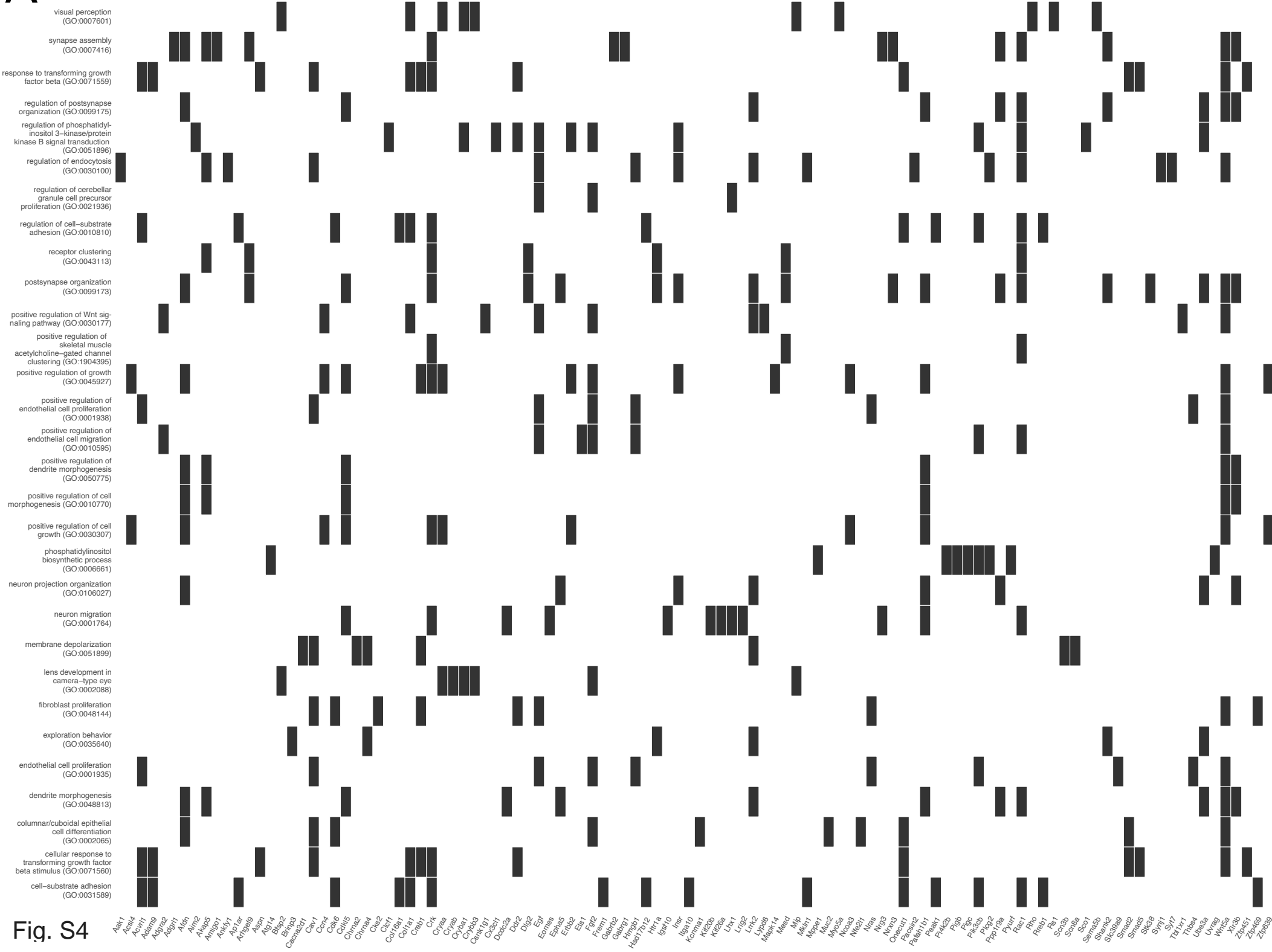

Fig. S4

Supplement: Supplemental_Figure_S4_Heatmap_for_Q344ter_GO_terms_and_genes_WT_ddaf146 [file supplemental_figure_s4_heatmap_for_q344ter_go_terms_and_genes_wt_ddaf146.pdf]

**A**

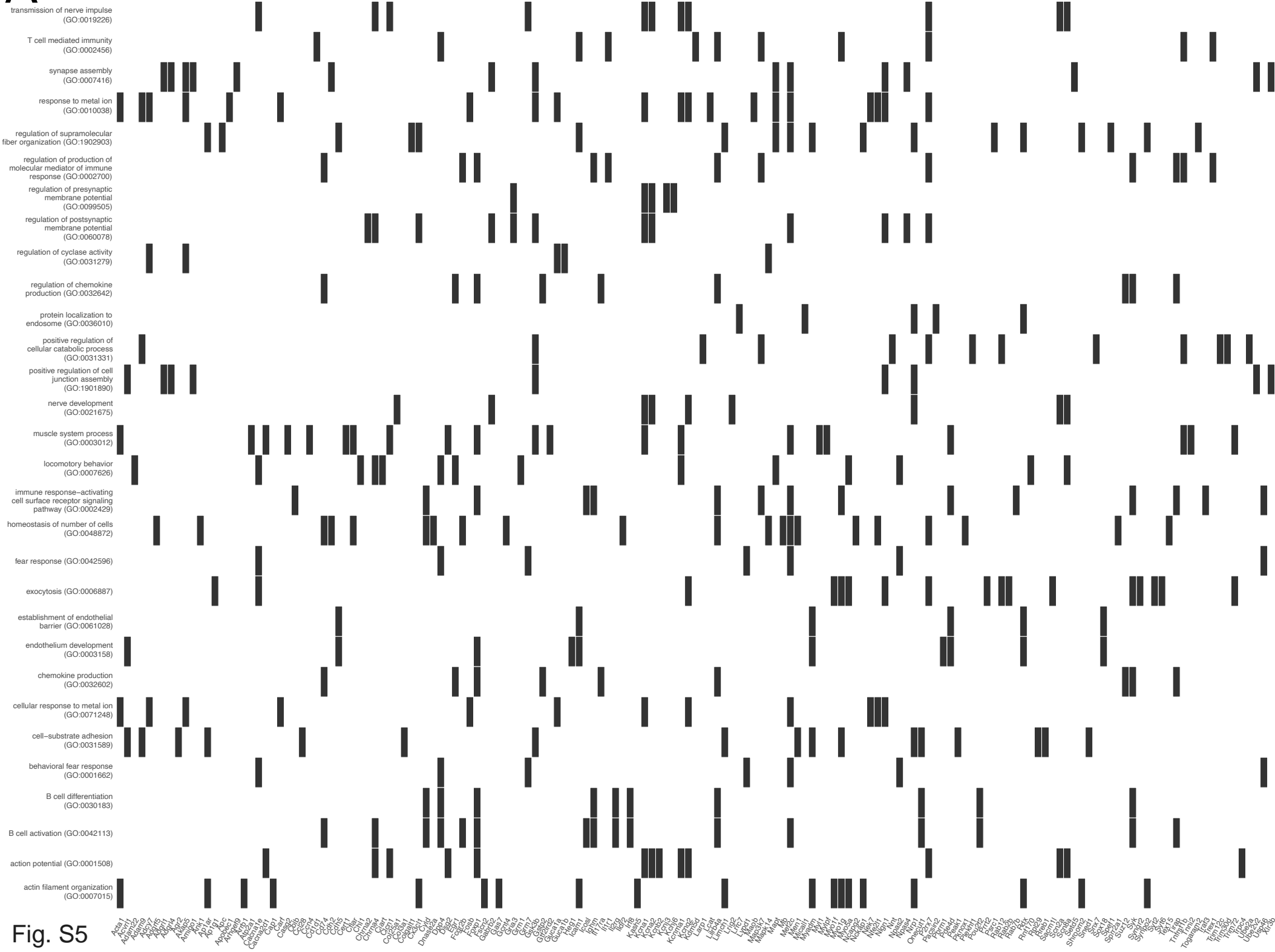

Fig. S5

Supplement: Supplemental_Figure_S5_Heatmap_for_Q344ter_GO_terms_and_genes_Q344ter_ddaf146 [file supplemental_figure_s5_heatmap_for_q344ter_go_terms_and_genes_q344ter_ddaf146.pdf]

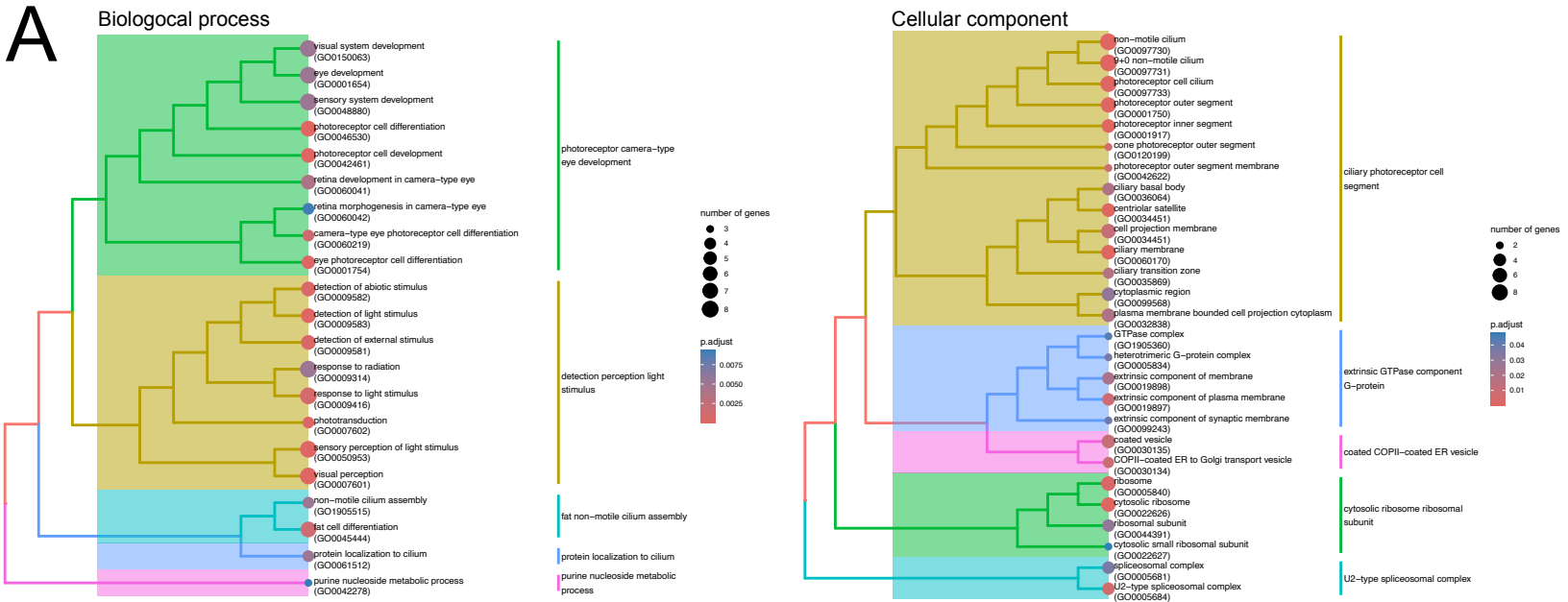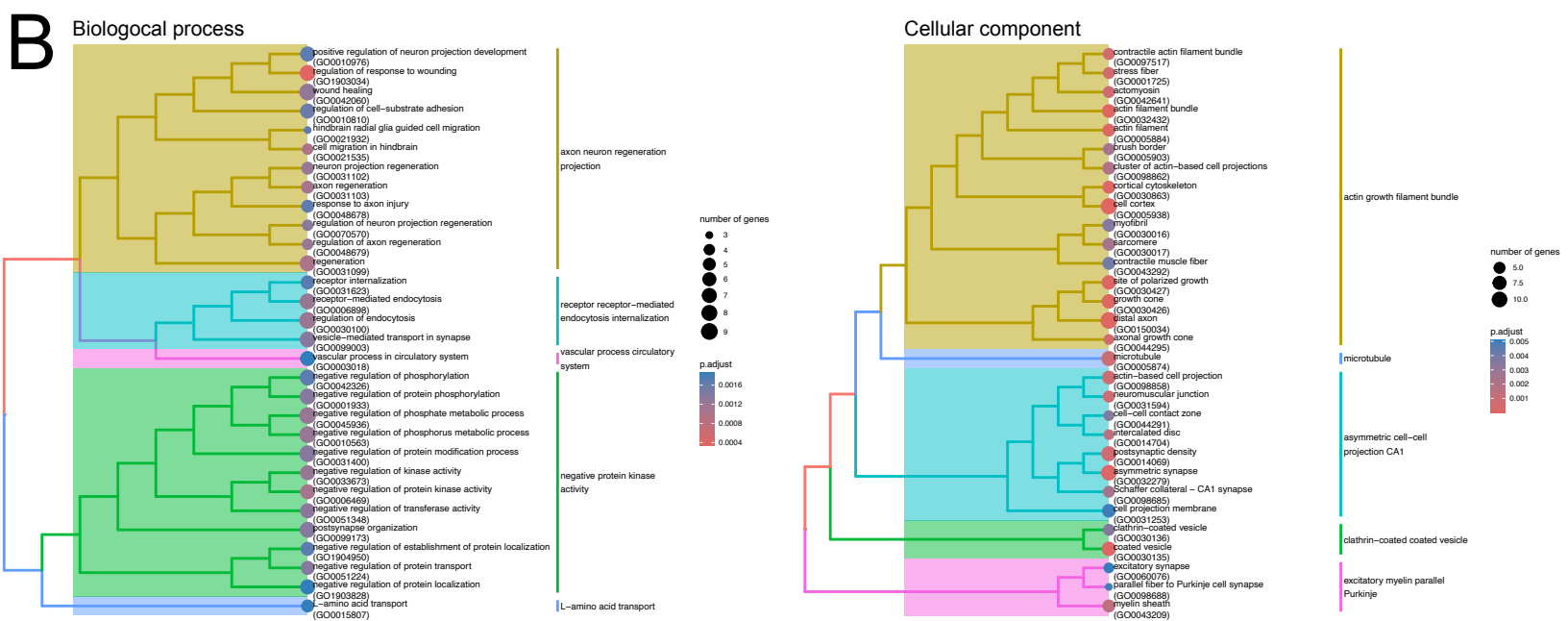

Supplemental Figure S6

Supplement: Supplemental_Figure_S6_Q344X_Multiomics_Tree_plots_071525_ddaf146 [file supplemental_figure_s6_q344x_multiomics_tree_plots_071525_ddaf146.pdf]
