# Supplementary material for: Transcriptional downregulation of rhodopsin is associated with desensitization of rods to light-induced damage in a murine model of retinitis pigmentosa
Source: Hum Mol Genet. 2025 Oct 7;34(22):1884–901. doi: 10.1093/hmg/ddaf146 (PMC12581828; doi:10.1093/hmg/ddaf146)
Supplement: HMG-2025-OA-00277_Takita_Supplementary_legend_ddaf146 [file hmg-2025-oa-00277_takita_supplementary_legend_ddaf146.docx]

**Supplemental Figure S1. *Rho* mRNA is downregulated in P35 *Rho^P23H/+^* retinas.**

(A) RNA expression levels were plotted to compare P35 wild-type and *Rho^P23H/+^* mouse retinas reared under standard cyclic 200 lux conditions. A total of 811 genes (shown in red dots) are differentially expressed with 2-fold change (log2-transformed change of < -1 or > 1) and q-value of < 0.05 thresholds. *Rho* was the second most abundantly expressed gene in wild-type retinas, and was downregulated by 75.3% in *Rho^P23H/+^* retinas. The dark gray shade indicates less than a 20% decline in mRNA expressions. The light gray shade indicates less than a 50% decline in mRNA expressions.

**Supplemental Figure S2. Associations between Gene Ontology (GO) terms for biological processes and specific genes for P35 *Rho^Q344X/+^* retinas in comparison to wild-type retinas under standard cyclic 200 lux light condition.**

(A) Gene names are shown on the x-axis and significantly enriched GO terms are shown on the y-axis. Black rectangles show their corresponding matches and visualize the associations between GO terms and protein names.

**Supplemental Figure S3. Comparison of RNA-seq between P35 wild-type and *Rho^Q344X/+^* retinas identifies that inflammation-related genes are upregulated in *Rho^Q344X/+^* retinas.**

(A) Pathway analyses for GO terms using the mRNA expression with 2-fold change are shown. Significantly changed pathways (up to 20) are shown for biological process, cellular component, and molecular function. The size of each dot within the plots represents the level of protein enrichment (Count), and the color coding indicates statistical significance (p.adjust), as shown on the right side of each panel.

(B) Networks illustrating the output of the hypergeometric test. The pathway analysis was based on gene enrichment. The size of each dot (Count) represents the level of gene enrichment (number of genes in each category), and the color coding shows statistical significance (p.adjust), as indicated at the bottom left corner.

(C) Significantly altered GO term pathways are grouped based on their functional or semantic similarities and shown in tree plots, reflecting higher-order semantic similarity-based broader biological themes for biological process (upper left, adjusted *p* < 0.005), cellular component (upper right, adjusted *p* < 0.01), and molecular function (bottom, adjusted *p* < 0.05). The size of each dot within the plots represents the level of protein enrichment (Count), and the color coding indicates statistical significance (p.adjust), as shown on the right side of each panel.

**Supplemental Figure S4. Associations between GO terms for biological processes and specific genes for P35 wild-type retinas reared under dark condition.**

(A) Gene names are shown on the x-axis and significantly enriched GO terms are shown on the y-axis. Black rectangles show their corresponding matches and visualize the associations of enriched GO terms and protein names.

**Supplemental Figure S5. Associations between GO terms for biological processes and specific genes for P35 *Rho^Q344X/+^* retinas reared under dark condition.**

(A) Gene names are shown on the x-axis and significantly enriched GO terms are shown on the y-axis. Black rectangles show their corresponding matches and visualize the associations of enriched GO terms and protein names.

**Supplemental Figure S6. Multiomics pathway analyses between P35 wild-type and *Rho^Q344X/+^* retinas reveal distinct proteomic alterations.**

(A) In *Rho^Q344X/+^* mice at P35, GO term pathways that showed significant protein‑level downregulation relative to wild‑type, but were unaltered in RNA‑seq data, were grouped based on functional or semantic similarity. These pathways are visualized as tree plots grouped by semantic similarity-based broader biological themes, with biological process terms on the left (adjusted *p* < 0.01) and cellular component terms on the right (adjusted *p* < 0.05). Dot size reflects the number of enriched proteins (Count), while color indicates statistical significance (adjusted p-value, p.adjust), as shown in the color scale on the right of each panel.

(B) In *Rho^Q344X/+^* mice at P35, GO term pathways that showed significant protein‑level upregulation relative to wild‑type, but were unaltered in RNA‑seq data, were grouped based on functional or semantic similarity. These pathways are visualized as tree plots grouped by semantic similarity-based broader biological themes, with biological process terms on the left (adjusted *p* < 0.01) and cellular component terms on the right (adjusted *p* < 0.05). Dot size reflects the number of enriched proteins (Count), while color indicates statistical significance (adjusted p-value, p.adjust), as shown in the color scale on the right of each panel.
